# Joint Association of Screen Time and Physical Activity with Cardiometabolic Risk Factors in a National Sample of Iranian Adolescents: The CASPIANIII Study

**DOI:** 10.1371/journal.pone.0154502

**Published:** 2016-05-11

**Authors:** Ramin Heshmat, Mostafa Qorbani, Amir Eslami Shahr Babaki, Shirin Djalalinia, Asal Ataei-Jafari, Mohammad Esmaeil Motlagh, Gelayol Ardalan, Tahereh Arefirad, Fatemeh Rezaei, Hamid Asayesh, Roya Kelishadi

**Affiliations:** 1 Chronic Diseases Research Center, Endocrinology and Metabolism Population Sciences Institute, Tehran University of Medical Sciences, Tehran, Iran; 2 Department of Community Medicine, School of Medicine, Alborz University of Medical Sciences, Karaj, Iran; 3 Endocrinology and Metabolism Research Center, Endocrinology and Metabolism Clinical Sciences Institute, Tehran University of Medical Sciences, Tehran, Iran; 4 Non-communicable Diseases Research Center, Endocrinology and Metabolism Population Sciences Institute, Tehran University of Medical Sciences, Tehran, Iran; 5 Development of Research & Technology Center, Deputy of Research and Technology, Ministry of Health and Medical Education, Tehran, Iran; 6 Department of Nutrition, Science and Research Branch, Islamic Azad University, Tehran, Iran; 7 Department of Pediatrics, Ahvaz Jundishapur University of Medical Sciences, Ahvaz, Iran; 8 Department of Exercise Physiology, Science and Research Branch, Islamic Azad University, Tehran, Iran; 9 Department of Social Medicine, Medical School, Jahrom University of Medical Sciences, Jahrom, Iran; 10 Department of Medical Emergencies, Qom University of Medical Sciences, Qom, Iran; 11 Pediatrics Department, Child Growth and Development Research Center, Research Institute for Primordial Prevention of Non Communicable Disease, Isfahan University of Medical Sciences, Isfahan, Iran; Mexican Social Security Institute, MEXICO

## Abstract

Metabolic syndrome (MetS) and its contributing factors are considered important health problems in the pediatric age group. This study was designed to assess the joint association of ST and PA with cardiometabolic risk factors among Iranian adolescents. A representative sample of 5625 (50.2% boys) school students with a mean age of 14.73 (SD: 2.41) were selected through multistage random cluster sampling method from urban and rural areas of 27 provinces in Iran. ST and PA were assessed by self-administered validated questionnaires. Anthropometric measures (height, weight and waist circumference (WC)) and MetS components (abdominal obesity, elevated blood pressure (BP), low high-density lipoprotein cholesterol (HDL-C), elevated triglycerides (TG) and high fasting blood sugar (FBG)) were measured according to standardized protocols. MetS was defined according to the Adult Treatment Panel III criteria modified for the pediatric age group. Moreover, elevated total cholesterol (TC), elevated low-density lipoprotein cholesterol (LDL-C), and generalized obesity were considered as other cardiometabolic risk factors. Students with high ST levels had significantly higher body mass index z-score (BMI z-score), WC, TG, LDL-C, and BP as well as lower HDL-C level; whereas those with high PA levels had significantly higher HDL-C levels as well as lower BMI z-score, TC, and BP. Adolescents with low PA/ high ST levels had significantly higher BMI, WC, LDL-C levels, as well as higher SBP and DBP compared to their other counterparts. In Multivariate model, joint effect of low PA/ high ST (compared to the high PA/low ST group) increased the odds of overweight, abdominal obesity and low HDL-C and decreased the odds of elevated TC. The findings of this study showed that joint association of high ST and low PA have direct association with abdominal obesity, overweight and low HDL-C and indirect association with elevated TC.

## Introduction

Metabolic syndrome (MetS) is defined as a cluster of risk factors for various non-communicable diseases, namely diabetes mellitus (DM) and cardiovascular diseases (CVD) [1].

Pediatric MetS is no more limited to industrial societies; recent studies have demonstrated that MetS is rapidly increasing in Iranian adolescents [2–9] similar to many other developing and Western countries [10–17]; it has been documented even in those with normal weight [18]. Some studies have explored the notion of persistence of MetS and other cardiometabolic risk factors into adulthood [1,7,19]. Notably, this combination of risk factors, rather than each of them, contributes to additional risks, beyond the sum of risks attributed to each individual risk factors [20].

CVDs and their major risk factors become highly prevalent worldwide and it is of special concern in the Middle East [21]. The evidences of epidemiological studies show that geographical differences and ethnicity are two main factors affecting the prevalence of cardiometabolic risk factors in different populations [22,23]. The most frequent metabolic risk factors in Iranian pediatric population are low levels of HDL-C, hypertriglyceridemia and overweight, respectively [24].

Sedentary lifestyle including low physical activity (PA) and prolonged screen time (ST)are considered as one of the major health problems in the pediatric population of developing and developed countries[25–27]. Previous studies have reported a considerably high prevalence of low PA and prolonged ST in Iranian adolescents [28,29].

Various studies have examined the associations of ST, i.e. leisure time spent in front of television (TV) or computer, and PA with cardiometabolic risk factors [30–33]. It is shown that changes in life style including; reducing routine daily activities of children may contribute to lower PA in this age group [34]. Some controversies exist about the associations of PA and ST with some components of MetS [35]. Some studies found that ST and PA have weak correlations [36–38]; whereas some other studies reported that lower energy expenditure and PA might mostly contribute to higher prevalence of metabolic abnormalities [35, 39].A study by Mitchell *et al*. argued that sedentary behavior has neglected effect on obesity; and suggested that combined effects PA and ST should be studied [40].

Therefore, the present study was designed to examine the associations of the ST, PA and their joint association with MetS and cardiometabolic risk factors in Iranian adolescents.

## Material and Methods

A national representative sample of third survey of the school-based surveillance system entitled “Childhood and Adolescence Surveillance and PreventIon of Adult Non-communicable Disease” (CASPIAN) study (2009–2010). The aim and methods of mentioned study is described previously [41].

Participants were 5625 students, aged 10–18 years, who were selected via multistage random cluster sampling method from urban and rural areas of 27 provinces in Iran. Data for some variables was missing. Based on the protocol of the study, the stratification of eligible schools was followed using the information bank of the Ministry of Health and Medical Education. In the next step sampled schools were selected randomly. In later step, in each of selected schools, the sampling of students was randomly. In data gathering phase, all processes of examinations with calibrated instruments and recording of information in validated checklists were designed and conducted under the standard protocol by trained health care professional teams.

The study was approved by the ethical committee of Tehran and Isfahan University of Medical Sciences and other relevant national regulatory organizations (Ministry of Health and Ministry of Education). Participation in the study was voluntarily. Sampling and examinations were begun after complete introducing of project and explanation of the study’s protocols for students and their parents. Oral assent was obtained from participants and written informed consent from their parents or legal guardians.

### Clinical and Laboratory Measurements

Height and weight were measured, according to standardized protocols, without shoes and with light clothing to the nearest 0.1 unit of measure (cm for height and kg for weight). Body mass index (BMI) was calculated from weight and height [BMI = weight (kg) ⁄height (m2)] [4, 42]. Waist circumference (WC) was measured over skin, midway between the lower border of the rib margin and the iliac crest at the end of normal expiration, to the nearest 0.1 cm. Both of WC and height were measured using a non-elastic tape. Abdominal obesity was defined as waist to height ratio (WHtR) more than 0.5[43].

Systolic blood pressure (SBP) and diastolic blood pressure (DBP) were measured, using a standardized mercury sphygmomanometer, on the right arm after a 15-minute rest in a sitting position. The first and fifth Korotkoff sounds were respectively recorded as systolic and diastolic blood pressure. The mean of the two measurements was considered as the subject’s blood pressure.

For each of participants, a blood sample was drawn between 7:00 and 9:00 AM after 12 to 14 hours overnight fasting. Blood samples were delivered to the laboratory on the same day. Fasting blood glucose (FBG), total cholesterol (TC), triglycerides (TG), high density lipoprotein-cholesterol (HDL-C), and low-density lipoprotein cholesterol (LDL-C) were measured enzymatically by auto-analyzers. HDL-C was determined after dextran sulfate-magnesium chloride precipitation of non-HDL-C [44].

As the highest quality of data was critical to the success of our multi-center data gathering, the different levels of quality assurance and control were exactly considered by Data and Safety Monitoring Board (DSMB) of the project.

### Demographic Information

Demographic information was completed for all participant students in the sampled classes of the selected schools through an interview with parents or child. Family based characteristics including: family history of chronic diseases (hypertension, dyslipidemia, diabetes, and obesity), parental level of education (the highest total years of schooling), possessing a family private car and type of home (rented/owned), dietary behaviors, PA, and sedentary lifestyle.

### Definition of Terms

#### Cardiometabolic risk factors

If students had at least three of the following criteria according to Adult Treatment Panel III (ATP III) criteria modified for children and adolescents, were considered as having MetS. The modified criteria for children and adolescents have been defined as follow: Abdominal obesity as waist to height ratio (WHtR) more than 0.5; Elevated BP: either systolic or diastolic BP ≥90th percentile for age, sex and height; Low HDL-C: HDL-C≤40 mg/dl (except in boys 15–19 years old that the cut off was <45 mg/dl); High TG: TG≥100 mg/dl) was taken as the 90th percentile value for age; High FBG: FBG levels of ≥100 mg/dl[45].Five criteria of MetS and TC, LDL-C, and general obesity were included in this study as cardiometabolic risk factors. High TC and LDL-C was defined according to the recent recommendation by the American Heart Association (TC≥200 mg/dl, LDL-C>110mg/dl [46, 47].

The overweight and general obesity definition provided by the Centers of Disease Control and Prevention (CDC) and the percentiles computed in the population studied were used for the classification of the adolescents as overweight (85–94th percentile) and obese (>95th percentile)[46].

#### Socioeconomic status (SES)

The method and variables, which was used for calculating SES was approved previously in the Progress in the International Reading Literacy Study (PIRLS) [48] Using principle component analysis (PCA) method variables including parental education, parents’ job, possessing private car, school type (public/private), and having personal computer in home were summarized in one main component. This main component was categorized into tertiles. The first tertile was defined as a low SES, second tertile as an intermediate and third tertile as a high.

#### ST and PA

In this study, the ST behavior of the adolescents was assessed through the questionnaire that asked the child to report the average number of hours per day they spent watching TV/VCDs, personal computer (PC), or electronic games (EG) in week days and weekends. Based on that total cumulative spent time for ST was estimated. For the analysis of correlates of ST, according to the international ST recommendations, ST was categorized into two groups; less than 2 hours per day (Low), and 2 hours per day or more (High) [49, 50].

For leisure time PA, the information of past week was collected. Participants reported the weekly frequency of their leisure time PA outside the school. Having PA, considered as at least 30 minutes duration of daily exercises might lead to heavy sweating or large increases in breathing or heart rate. Therefore, we categorized weekly PA habits through available response choices as follow; none, 1–2 days, 3–6 days, and every day. For statistical analysis, PA was categorized into low (0–2 days /week) and high (3–7 days/week) levels [51].

The joint associations of PA and ST were considered based on following possible mixed conditions: Low PA & Low ST, Low PA & High ST, High PA &Low ST, and High PA & High ST.

### Statistical Analyses

Quantitative variables are expressed as mean (standard deviation (SD)) and qualitative variables as number (percentage). Differences between means were investigated by T-test or ANOVA test (followed by Tukey’s post- hoc tests) and for categorized variables; the Pearson Chi-square test used to comparison the percentages. Logistic regression analyses were used to evaluate the joint association of PA and ST categories with odds of cardiometabolic risk factors. In Model I the joint association of PA/ST categories with cardiometabolic risk factors were assessed without adjustment. In Model II the association was adjusted for age and sex and in Model III, additionally family history and SES were adjusted in the model. In Model IV in addition to all variables in the Model III, BMI was adjusted in all abnormalities except obesity. In all models “high PA & low ST” group was considered as reference group because this combination according to the international ST recommendations has lowest risk of cardiometabolic risk factors compared to other combinations. Results of logistic regression are presented as odds ratio (OR) and 95% confidence interval (CI). In all analysis design of sampling (cluster sampling) were considered. Data was analyzed using "survey data method". All statistical analyses were performed using programs available in the STATA version 10. A *p-value* of less than 0.05 was considered as statistically significant.

## Results

The participants of this national study were 5625 students (50.2% boys, 49.8% girls) with a mean age of 14.73 (SD: 2.41). A comparison of baseline characteristics is presented in Table 1. It shows that boys had higher BMI z-score (*p< 0*.*01*), whereas girls had greater height, weight and WC measurements (*p< 0*.*001*). Considering the combination of times spent on watching TV and working with the computer, most of the adolescents had low ST levels (54.1% of all participants) which was statistically significant between both sexes (56.8% of boys and 51.3% of girls) (*p< 0*.*001*); boys and girls spent equal amounts of time in front of TV, while for working with computer, boys spent more time than girls which was statistically significant (*p< 0*.*001*). Distribution of PA levels among boys and girls pointed out a significant difference among those PA level (*p< 0*.*001*); the majority of participants of both genders and the overall sample had low PA levels (88.4% of boys, 81.3% of girls and 84.9% of all), while more girls were highly active. Regarding joint association of ST and PA there was a statistically significant difference between boys and girls. In this regard, the combination of low ST and low PA in boys was significantly more prevalent than in girls (*p< 0*.*001*) (Table 1).

**Table 1:** Demographic characteristics and anthropometric measures according to sexes: The CASPIAN III study.

	Boys	Girls	Total	*p value*
Age (year)	14.69 (2.45)	14.76(2.37)	14.73(2.41)	0.24
Height (cm)	151.80(11.64)	156.54(15.59)	154.16(13.95)	<0.001*
Weight (Kg)	45.93(13.21)	48.42(16.53)	47.17(15.00)	<0.001*
BMI z-score	0.46 (1.0)	-0.46 (1.0)	0(1.0)	0.001*
WC (cm)	67.60(22.18)	69.85(18.99)	68.72(20.69)	<0.001*
WHtR	0.44 (0.14)	0.45(0.12)	0.44(0.12)	0.73
Obesity; n (%)	286 (10.1)	215 (7.7)	501 (8.9)	0.001*
Overweight; n (%)	265 (9.4)	186 (6.6)	451 (8.0)	<0.001*
Abdominal obesity; n (%)	415 (14.7)	471 (16.9)	886 (15.8)	0.02*
Father’s education; n (%)				0.48
<6y	1138 (41.4)	1153 (42.6)	2291 (42.0)	
6–9y	662 (24.1)	668 (24.7)	1330 (24.4)	
10–12y	685 (24.9)	626 (23.2)	1311 (24.0)	
>12y	263 (9.6)	257 (9.5)	520 (9.5)	
Mother’s education; n (%)				0.50
<6y	1489 (53.6)	1515 (55.6)	3004 (54.6)	
6–9y	580 (20.9)	548 (20.1)	1128 (20.5)	
10–12y	570 (20.5)	534 (19.6)	1104 (20.1)	
>12y	141 (5.1)	128 (4.7)	269 (4.9)	
ST Activity(hours/day)				
Watching TV; n (%)				0.65
<2	991 (35.6)	940 (35.0)	1931 (35.3)	
≥2	1790 (64.4)	1742 (65.0)	3532 (64.7)	
Working with computer; n (%)				<0.001*
<2	2372 (84.3)	2803 (76.5)	4453 (80.4)	
≥2	443 (15.7)	641 (23.5)	1084 (19.6)	
ST; n (%) ^¶^				<0.001*
Low	1576 (56.8)	1370 (51.3)	2946 (54.1)	
High	1199 (43.2)	1303 (48.7)	2502 (45.9)	
PA; n (%)^£^				<0.001*
Low	2491 (88.4)	2224 (81.3)	4715 (84.9)	
High	327 (11.6)	510 (18.7)	837 (15.1)	
Joint associations of PA & ST; n (%)				<0.001*
High PA & Low ST	208 (7.5)	262 (9.8)	470 (8.6)	
High PA & High ST	114 (4.1)	235 (8.8)	349 (6.4)	
Low PA & Low ST	1368 (49.3)	1107 (41.4)	2475 (45.5)	
Low PA & High ST	1083 (39.1)	1068 (40.0)	2151 (39.5)	
Family history; n (%)				
HTN	1134 (51.4)	1039 (47.7)	2173 (49.5)	0.02*
Dyslipidemia	909 (42.7)	859 (40.3)	1768 (41.5)	0.13
DM	831 (39.1)	717 (34.2)	1548 (36.7)	0.001*
Obesity	876 (42.8)	747 (37.1)	1623 (40.0)	<0.001*
Type of home; n (%)				0.63
Rented home	531 (19.5)	531 (20.0)	1062 (19.8)	
Owned home	2193 (80.5)	2119 (80.0)	4312 (80.2)	
Private car; n (%)				0.83
Yes	1373 (49.8)	1340 (49.5)	2713 (49.7)	
No	1382 (50.2)	1366 (50.5)	2748 (50.3)	
SES; n (%)				0.76
Low	527 (19.6)	525 (20.0)	1052 (19.8)	
Intermediate	1047 (38.9)	1038 (39.5)	2085 (39.2)	
High	1115 (41.5)	1063 (40.5)	2178 (41.0)	

Data are means (SD) unless indicated otherwise.

BMI; Body Mass Index, WC; Waist Circumference, HTN; Hypertension, DM; Diabetes Mellitus, ST; Screen Time, PA; Physical Activity, SES; Socioeconomic status.

^¶^ Low ST: <2 hours/day; high ST: ≥2 hours/day

^£^Low PA: 0–2 days/week; high PA: 3–7 days / week

* Statistically significant

Table 2 presents the associations between cardiometabolic risk factors with ST, PA and joint association of PA and ST categories. Students with high ST levels had significantly higher BMI z-score, WC, TG, LDL and BP and lower HDL level; those with high PA levels had significantly lower BMI z-score, TC and BP and higher HDL levels. Regarding the joint association of PA and ST, we observed that mean of all cardiometabolic risk factors except WHtR, FBG and TG were significantly different across PA/ST levels. Results of post hoc test show that students with low PA and high ST levels had significantly higher BMI z-score, SBP and DBP, compared to the high PA and low ST levels; these individuals had the lowest serum HDL-C levels among all participants (*p< 0*.*05*).

**Table 2:** Mean (SD) of cardiometabolic values according to ST, PA and joint associations of ST and PA categories: The CASPIAN III study.

	ST ^¶^			PA ^£^			Joint association of PA and ST				
	Low	High	p-value	Low	High	p-value	High PA/ Low ST	High PA & High ST	Low PA & Low ST	Low PA/ High ST	P-value
BMI z-score	-0.07 (1.0)	0.09 (1.0)	<0.001*	0.01 (1.0)	- 0.07 (1.0)	0.03*	-0.15 (1.0)^a^	0.07 (1.0)^b^	-0.05 (1.0)^ab^	0.09 (1.0)^bc^	<0.001*
WC (cm)	67.9 (22.8)	69.7 (18.5)	0.002*	68.8(20.4)	68.3(22.8)	0.03*	67.8(28.9)^ab^	69.4(10.7)^ab^	67.9(21.4)^b^	69.7(19.4)^a^	0.023*
WHtR	0.45 (0.15)	0.45(0.11)	0.96	0.44(0.13)	0.45 (0.15)	0.67	0.45(0.19)	0.44(0.05)	0.44(0.14)	0.45(0.11)	0.851
FBG (mg/dl)	87.8 (13.4)	87.5(14.6)	0.45	87.5(14.1)	88.6 (12.6)	0.06	88.0(12.5)	89.5(12.9)	87.8(13.6)	87.2(14.8)	0.064
TG (mg/dl)	91.4 (38.9)	94.2(42.8)	0.02*	92.8 (41.1)	91.9(39.8)	0.59	90.6(38.7)	93.8(41.6)	91.6(39.0)	94.3(43.0)	0.137
HDL-C (mg/dl)	47.1 (14.5)	45.3(14.3)	<0.001*	45.9(14.1)	48.5(14.9)	<0.001*	47.9(14.6)^a^	49.4(15.7) ^a^	46.9(14.5) ^a^	44.7(13.8)^b^	<0.001*
TC (mg/dl)	148.2 (31.4)	149.1(32.3)	0.36	151.3(33.3)	147.9(31.4)	0.01*	151.7(34.1)^a^	151.4(32.5)^ab^	147.5(30.8)^b^	148.7(32.2)^ab^	0.035*
LDL-C (mg/dl)	82.9 (26.9)	85.7(27.8)	0.005*	84.2(27.4)	84.1(27.3)	0.91	84.0 (27.6)^ab^	84.1(27.1)^ab^	82.7(26.8)^b^	85.9(27.9)^a^	0.025*
SBP (mmHg)	102.6 (13.8)	103.9(14.0)	<0.001*	103.3(13.9)	102.2(14.1)	0.04*	101.2(14.1)^a^	103.6(13.9)^ab^	102.8(13.7)^a^	104.0(14.1)^b^	0.001*
DBP (mmHg)	65.3 (10.8)	66.2(10.6)	0.001*	65.8(10.6)	64.8(11.2)	0.01*	64.6(11.5)^a^	65.2(10.9)^ab^	65.4 (10.6)^a^	66.4(10.6)^b^	0.001*

Data are mean(SD),BMI; Body Mass Index, WC; Waist Circumference, WHtR; Waist to Height Ratio, FBS; Fasting Blood Sugar, TG; Triglycerides, HDL-C; High Density Lipoprotein-Cholesterol, TC; Total Cholesterol, LDL-C; Low-Density Lipoprotein Cholesterol, SBP; Systolic Blood Pressure; DBP; diastolic Blood Pressure, ST; Screen Time, PA; Physical Activity.

For joint association of PA and ST, within rows, means with different superscript letters are significantly different (by Tukey’s post hoc tests).

^¶^ Low ST: <2 hours/day; high ST: ≥2 hours/day

^£^Low PA: 0–2 days/week; high PA: 3–7 days / week

* Statistically significant

Table 3 shows the prevalence of cardiometabolic risk factors according to ST and PA levels categories and their joint association. Individuals with high ST levels had a higher prevalence of having elevated serum TG and LDL levels, being overweight or having lower serum HDL levels; although there was a significant difference between the prevalence of different MetS components in low and high ST levels, prevalence of MetS was similar in the two categories. Considering PA levels, participants with high PA levels had a lower prevalence of low HDL levels (27.2% versus 36.4%) or elevated serum TC levels (5.3% versus 8.7%). When accounting for the joint association of PA and ST, participants with low PA/ high ST levels had the highest prevalence of low serum HDL levels; those with high PA/ low ST levels had the highest prevalence of elevated serum TC levels (*p< 0*.*01*) (Table 3).

**Table 3:** Prevalence of cardiometabolic risk factors according to ST, PA and joint associations of ST and PA categories: The CASPIAN III study.

	ST			PA			Joint association of ST and PA				
	Low	High	*p value*	Low	High	*p value*	High PA/ Low ST	High PA & High ST	Low PA & Low ST	Low PA/ High ST	*p value*
Abdominal obesity	445 (15.1)	416 (16.7)	0.17	756 (16.1)	123 (14.7)	0.33	66 (14.0)	56 (16.0)	379 (15.3)	360 (16.8)	0.384
Elevated SBP	96(3.5)	94 (4.0)	0.37	168 (3.8)	28 (3.6)	0.9	12 (2.8)	16 (5.0)	84 (3.7)	77 (3.8)	0.464
Elevated DBP	81 (2.9)	76 (3.2)	0.57	128 (2.9)	29 (3.7)	0.26	16 (3.6)	13 (3.9)	65 (2.8)	63 (3.1)	0.582
Elevated BP	140 (5.3)	141 (6.2)	0.16	236 (5.5)	47 (6.2)	0.44	23 (5.4)	24 (7.7)	117 (5.2)	117 (6.0)	0.321
Elevated TG	172(7.1)	187 (9.2)	0.01*	310 (8.1)	54 (7.8)	0.88	31 (7.9)	22 (7.7)	141 (7.0)	165 (9.4)	0.051
Elevated FBG	356(15.0)	317(16.2)	0.27	561(15.0)	119(17.4)	0.12	64 (16.5)	53 (19.0)	291 (14.6)	264 (15.8)	0.255
Low HDL-C	694(33.5)	651(36.9)	0.03*	1210 (36.4)	159 (27.2)	<0.001*	98 (29.8)	59 (24.4)	596 (34.3)	592 (38.9)	<0.001*
Elevated TC	139 (5.7)	127 (6.0)	0.61	207 (5.3)	60 (8.7)	.001*	37 (9.4)	23 (8.1)	102 (5.0)	104 (5.7)	0.002*
Elevated LDL-C	130 (5.0)	130 (6.9)	0.03*	220 (5.9)	35 (5.0)	0.45	20 (4.9)	20 (5.5)	105 (5.0)	124 (7.1)	0.117
General obesity	257 (8.7)	230 (9.2)	0.57	427 (9.1)	70 (8.4)	0.56	39 (8.3)	31 (8.9)	218 (8.8)	199 (9.3)	0.908
Overweight	217 (7.4)	224 (9.0)	0.04*	390 (8.3)	60 (7.2)	0.30	30 (6.4)	29 (8.3)	187 (7.6)	195 (9.1)	0.135
Number of Mets components			0.04*			0.46					0.301
0	840 (46.2)	630 (40.9)		1259 (43.3)	241 (46.6)		143 (48.3)	92 (43.6)	697 (45.8)	537 (40.4)	
1	695 (38.2)	631 (40.9)		1156 (39.8)	195 (37.7)		111 (37.5)	80 (37.9)	583 (38.3)	551 (41.4)	
2	216 (11.9)	213 (13.8)		367 (12.6)	67 (13.0)		34 (11.5)	33 (15.6)	182 (12.0)	180 (13.5)	
3	58 (3.2)	57 (3.7)		105 (3.6)	11 (2.1)		6 (2.0)	5 (2.4)	52 (3.4)	52 (3.9)	
4	10 (0.6)	11 (0.7)		18 (0.6)	3 (0.6)		2 (0.7)	1 (0.5)	8 (0.5)	10 (0.8)	
Having MetS	68 (3.7)	68 (4.4)	0.34	123 (4.2)	14 (2.7)	0.11	8 (2.7)	6 (2.8)	60 (3.9)	62 (4.7)	0.321

Data are N (%), BP; Blood Pressure, TG; Triglycerides, FBG; Fasting Blood Glucose, HDL-C; High Density Lipoprotein-Cholesterol, TC; Total Cholesterol, LDL-C; Low-Density Lipoprotein Cholesterol, MetS; Metabolic Syndrome, ST; Screen Time, PA; Physical Activity.

Abdominal obesity: WHtR>0.05; Elevated SBP: systolic BP>90th adjusted by age, sex and height; Elevated DBP: diastolic BP>90th adjusted by age, sex and height; Elevated BP: either systolic or diastolic BP>90th adjusted by age, sex and height; Elevated FBS:>100 mg/dl; Elevated TG:> = 100 mg/dl; Low HDL: <50 mg/dl (except in boys 15–19 years old, that cut-off was <45 mg/dl); Elevated TC:>200 mg/dl; Elevated LDL:>110 mg/dl; Overweight: BMI:85th-95th; Obesity: BMI > 95th; Having MetS: having at least three criteria according to modified ATP III criteria; Low ST: <2 hours/day; high ST: ≥2 hours/day; Low PA: 0–2 days/week; high PA: 3–7 days / week.

* Statistically significant

Table 4 illustrates the comparison of joint effects of PA/ ST levels on cardiometabolic risk factors, against being highly physically active and having low ST levels (high PA/ low ST), using different logistic regression models. Only those with low PA levels had significantly different risks of having certain cardiometabolic risk factors; individuals with low PA/ high ST levels had their odds of being overweight and having abdominal obesity and low HDL-C increased by 73%, 48% and 40–50%, respectively. Their odds of having elevated serum TC levels were decreased 39–42%, based on the regression model. Participants with low PA/ low ST levels had just decreased odds of having elevated serum TC levels (50–52%) (Table 4).

**Table 4:** Odds ratios (95% CI) for cardiometabolic risk factors according to joint associations of ST and PA categories: The CASPIAN III study.

		Joint association of ST and PA						
		High PA & Low ST	High PA & High ST		Low PA & Low ST		Low PA & High ST	
			OR	CI	OR	CI	OR	CI
Abdominal obesity								
Model I		1	1.170	0.795–1.722	1.108	0.835–1.469	1.234	0.929–1.639
Model II		1	1.133	0.769–1.670	1.121	0.844–1.489	1.229	0.923–1.636
Model III		1	1.204	0.731–1.984	1.283	0.892–1.846	1.485*	1.029–2.144
Elevated BP								
Model I		1	1.455	0.805–2.629	0.969	0.612–1.534	1.115	0.704–1.766
Model II		1	1.208	0.664–2.199	0.906	0.569–1.444	0.944	0.592–1.506
Model III		1	1.202	0.582–2.483	1.063	0.613–1.844	0.991	0.567–1.732
Model IV		1	1.048	0.499–2.205	1.018	0.580–1.784	0.897	0.507–1.586
Elevated FBG								
Model I		1	1.191	0.797–1.780	0.871	0.648–1.171	0.949	0.704–1.280
Model II		1	1.127	0.752–1.689	0.913	0.678–1.231	0.971	0.717–1.314
Model III		1	1.072	0.678–1.695	0.829	0.595–1.155	0.918	0.656–1.286
Model IV		1	1.063	0.672–1.682	0.828	0.594–1.153	0.912	0.651–1.278
Elevated TG								
Model I		1	0.981	0.555–1.732	0.876	0.584–1.313	1.218	0.816–1.817
Model II		1	0.952	0.538–1.685	0.845	0.563–1.270	1.157	0.772–1.733
Model III		1	0.663	0.315–1.395	0.867	0.541–1.388	1.091	0.680–1.750
Model IV		1	0.586	0.274–1.250	0.811	0.500–1.315	0.984	0.605–1.598
Low HDL-C								
Model I		1	0.760	0.521–1.108	1.228	0.950–1.587	1.502*	1.161–1.944
Model II		1	0.709	0.486–1.036	1.187	0.917–1.536	1.400*	1.078–1.817
Model III		1	0.766	0.492–1.193	1.219	0.907–1.640	1.311	0.971–1.771
Model IV		1	0.748	0.480–1.165	1.218	0.906–1.639	1.297	0.960–1.753
Elevated TC								
Model I		1	0.842	0.489–1.452	0.502*	0.339–0.744	0.584*	0.394–0.864
Model II		1	0.896	0.518–1.549	0.499*	0.336–0.741	0.598*	0.401–0.891
Model III		1	0.956	0.506–1.808	0.500*	0.318–0.788	0.647	0.409–1.023
Model IV		1	0.908	0.479–1.720	0.487*	0.309–0.768	0.615*	0.388–0.976
Elevated LDL-C								
Model I		1	1.134	0.486–2.644	1.039	0.567–1.904	1.501	0.826–2.727
Model II		1	1.243	0.531–2.910	1.067	0.580–1.963	1.645	0.898–3.011
Model III		1	1.583	0.557–4.501	1.200	0.558–2.584	1.991	0.933–4.248
Model IV		1	1.542	0.542–4.389	1.192	0.554–2.566	1.958	0.917–4.180
Overweight								
Model I		1	1.329	0.782–2.259	1.199	0.805–1.786	1.462	0.982–2.177
Model II		1	1.400	0.822–2.384	1.163	0.779–1.736	1.452	0.972–2.171
Model III		1	1.577	0.813–3.058	1.224	0.743–2.017	1.773*	1.075–2.923
General obesity								
Model I		1	1.077	0.658–1.764	1.067	0.748–1.524	1.127	0.787–1.613
Model II		1	1.160	0.707–1.904	1.073	0.750–1.535	1.181	0.822–1.697
Model III		1	1.222	0.629–2.378	1.292	0.803–2.079	1.418	0.874–2.302
Having MetS								
Model I		1	1.054	0.360–3.083	1.477	0.699–3.123	1.760	0.834–3.717
Model II		1	0.898	0.306–2.638	1.410	0.664–2.995	1.557	0.733–3.310
Model III		1	0.991	0.260–3.782	1.599	0.623–4.101	1.571	0.606–4.074
Model IV		1	0.737	0.180–3.023	1.563	0.569–4.292	1.536	0.552–4.274

BP; Blood Pressure, TG; Triglycerides, FBG; Fasting Blood Glucose, HDL-C; High Density Lipoprotein-Cholesterol, TC; Total Cholesterol, LDL-C; Low-Density Lipoprotein Cholesterol, MetS; Metabolic Syndrome, ST; screen time, PA; physical activity.

Abdominal obesity: WHtR>0.05; Elevated BP: either systolic or diastolic BP>90th adjusted by age, sex and height; Elevated FBS:>100 mg/dl; Elevated TG:> = 100 mg/dl; Low HDL: <50 mg/dl (except in boys 15–19 years old, that cut-off was <45 mg/dl); Elevated TC:>200 mg/dl; Elevated LDL:>110 mg/dl; Overweight: BMI:85th-95th; Obesity: BMI > 95th; Having MetS: having at least three criteria according to modified ATP III criteria; Low ST: <2 hours/day; high ST: ≥2 hours/day; Low PA: 0–2 days/week; high PA: 3–7 days / week.

Model I: crude model; Model II is adjusted for age and gender; Model III adjusted additionally for family history and socio-economic status; Model IV is adjusted additionally for BMI in all abnormalities except obesity.

* Statistically significant

## Discussion

The current findings on the joint effects of PA and ST levels showed that adolescents with low PA/high ST levels had the higher BMI z-score, WC, LDL-C, SBP, and DBP, as well as the lower HDL-C levels compared to other combinations of PA/ST. In addition, participants with high PA/low ST had the highest mean serum levels of total cholesterol amongst other counterparts, which could be explained by high levels of HDL-C in this group. To the best of our knowledge, this is the first study in Middle Eastern adolescents that reports the combined associations of ST and PA with the odds of having cardio-metabolic risk factors.

Considering the importance and priority of problem, epidemiological aspects of cardiometabolic risk factors become one of the most attractive domain of health research [1,2,39, 46]. Highest prevalence of pediatric metabolic risk factors and MetS of developing countries are reported from the Eastern Europe and the Middle East, and the lowest one from India and Sri Lanka [52].

Our findings are consistent with some previous studies conducted on the joint effect of PA and ST or sedentary behaviors on BMI in Japanese [53], Australian [54], and US adults [55]. Our findings about joint association of PA and ST with cardiometabolic risk factors are contrary to Drenowatz et al. study [36]. Their study reported no significant association between joint effect of PA and ST with cardiometabolic risk factors in 10-year-old children; however, in that study, in line with our results low PA and high ST were related to a higher CVD risk score [36]. Some studies emphasized that higher activity levels were associated with a healthier diet and lower ST indicating an overall healthier lifestyle of this subgroup [33].

The current findings show that adolescents with low PA/ high ST were about 1.5 times more likely to have low levels of HDL-C, with approximately 40%lower risk of elevated TC compared to those with high PA/ low ST. After adjustment for confounding factors, these participants were 1.5 times more likely to have abdominal obesity, and 1.5 to 1.8 times more likely to be overweight than those in the high PA/ low ST group.

Some studies have reported that sufficient PA is associated with reduced cardiometabolic risk in children and adolescents [30, 56]. In a cross-sectional study in 1732 school children from Denmark, Estonia, and Portugal, negative association existed between PA and clustered cardiovascular risk [57]. Pooled data from 14 studies comprising 20871 children and adolescents, aged 4–18 years, also showed that moderate to vigorous PA might be associated with lower cardiometabolic risk factors, regardless of their amount of sedentary behaviors [58].

There is some evidences that the association between PA/ST and MetS differs according to sex and type of sedentary behaviors [59–60]. In the current study, sedentary behaviors included times spent for watching TV/VCDs and playing computer/electronic games, but other sedentary behaviors such as homework and motorized transport were not considered. A cohort study of Canadian children aged 8–10 years showed that not overall sedentary behaviors, but only ST was independently associated with WC and HDL-C [61]. In the present study, joint association of PA and ST was associated with sex. A cross-sectional study of French adults showed that the relationship of the MetS with ST and PA differs according to sex [60].

The absence of statistically significant associations between the PA/ST and elevated FBG, TG, and BP may be explained by the generally better status of these parameters or small number of adolescents with unfavorable levels of these risk factors.

One plausible mechanism responsible for the adverse association of sedentary behaviors on serum HDL-C is the suppression of the rate-limiting enzyme lipoprotein lipase activity in skeletal muscles. This is an enzyme necessary for HDL-C production and TG uptake [62, 63]. In addition to this physiologic mechanism, it is suggested that students eat unhealthy snacks more frequently while watching TV [63]. These findings suggest that lower BMI and WC, as well as improving HDL-C should be encouraged by highlighting the importance of increasing PA and reducing ST.

As one of the main strengths, present study benefited from a large national representative sample of Iranian adolescents. Moreover, it led exactly based on the World Health Organization- Global School-based student Health Survey (WHO-GSHS) protocol.

We also faced several limitations. First, the cross-sectional design of study which does not demonstrate the causality or specify the direction of causation between the cardiometabolic risk factors and PA/ST. Therefore, it may be that participants with a higher BMI do less PA and engage more extensively in sedentary behaviors like TV viewing. Future studies examining the prospective association of prolonged sitting on the risk of developing cardiometabolic risk factors would be warranted. Second, ST and PA data were based on self-reports that may be subject to recall bias. Finally, the possible contributing factors, as eating snacks or drinking soft drinks during ST, were not integrated into the study. Since some evidences show that association between PA/ST and MetS differs according to sex and type of sedentary behaviors, therefore stratify analysis according to sex and type of sedentary behaviors is suggested for future research.

A next step could be assessing the clustering of multiple life style risk factors associated with high ST levels in Iranian adolescents. This study used a large sample-size which was representative of all parts and strata of Iranian pediatric population. Moreover, as the basic practical evidences for interventional health programs, complementary researches on determinant of differences between boys and girls are recommend.

## Conclusion

The findings of this study showed that joint association of high ST and low PA have direct association with abdominal obesity, overweight and low HDL-C and indirect association with elevated TC. The current findings underscore the importance of reducing ST along with increasing PA for reducing the risk of developing cardiometabolic risk factors. Future public interventions are needed focusing on sedentary behaviors and PA from early life.
